# Neutrophil Extracellular Traps Induce Tissue-Invasive Monocytes in Granulomatosis With Polyangiitis

**DOI:** 10.3389/fimmu.2019.02617

**Published:** 2019-11-13

**Authors:** Mitsuhiro Akiyama, Markus Zeisbrich, Nour Ibrahim, Shozo Ohtsuki, Gerald J. Berry, Peter H. Hwang, Jörg J. Goronzy, Cornelia M. Weyand

**Affiliations:** ^1^Department of Medicine, Stanford University School of Medicine, Stanford, CA, United States; ^2^Department of Otolaryngology-Head and Neck Surgery, Stanford University, Stanford, CA, United States; ^3^Department of Pathology, Stanford University School of Medicine, Stanford, CA, United States

**Keywords:** granulomatosis with polyangiitis, NETosis, S100A9, cartilage destruction, bone destruction, matrix metalloproteinases, monocytes

## Abstract

**Objective:** Granulomatosis with polyangiitis (GPA) is a multi-organ vasculitic syndrome typically associated with neutrophil extracellular trap (NET) formation and aggressive tissue inflammation. Manifestations in head and neck (H&N) GPA include septal perforations, saddle-nose deformities, bony erosions of the orbital and sinus walls, middle ear damage and epiglottitis, indicative of bone, cartilage, and connective tissue destruction. Whether H&N-centric lesions engage disease pathways distinctive from the ischemic tissue damage in the lungs, kidneys, skin, and peripheral nerves is unknown. We have compared inflammatory responses triggered by neutrophilic NETs in patients with H&N GPA and systemic GPA (sGPA).

**Methods:** Neutrophils and monocytes were isolated from the peripheral blood of patients with H&N GPA, sGPA, and age/gender matched healthy individuals. Neutrophil NETosis was induced. NETs were isolated and cocultured with monocytes. Gene induction was quantified by RT-PCR, protein upregulation by flow cytometry. Tissue invasiveness of monocytes was measured in a 3D collagen matrix system. Expression of MMP-9 in tissue-residing macrophages was assessed by immunohistochemistry in tissue biopsies.

**Results:** Neutrophils from H&N GPA patients showed more intense NETosis with higher frequencies of netting neutrophils (*P* < 0.001) and release of higher amounts of NETs (*P* < 0.001). Isolated NETs from H&N GPA functioned as an inducer of danger-associated molecular patterns in monocytes; specifically, alarmin S100A9. NET-induced upregulation of monocyte S100A9 required recognition of DNA. S100A9 release resulted in the induction of metalloproteinases, including MMP-9, and enabled monocytes to invade into extracellular matrix. Anti-MMP-9 treatment attenuated the tissue invasiveness of monocytes primed with NETs from H&N GPA patients. MMP-9-producing macrophages dominated the tissue infiltrates in naso-sinal biopsies from H&N GPA patients.

**Conclusion:** Distinct disease patterns in GPA are associated with differences in NET formation and NET content. H&N GPA patients with midline cartilaginous and bony lesions are highly efficient in generating NETs. H&N GPA neutrophils trigger the induction of the alarmin S100A9, followed by production of MMP-9, endowing monocytes with tissue-invasive capabilities.

## Introduction

Granulomatosis with polyangiitis (GPA) is an autoimmune small to medium-sized vessel vasculitis characterized by acute and chronic tissue inflammation in the nose, the ear, the throat, the orbit, the lung, the skin, and the kidney (1, 2). Despite the systemic features of the disease, a localized form of GPA, dominated by ear, nose, throat, and eye lesions (H&N GPA), has been recognized as a distinct subtype (3–10). Clinical studies examining this limited form of GPA have reported a prevalence of 4–22% in different patient cohorts (11–13). Systemic GPA (sGPA) is considered a severe and potentially fatal disease, typically complicated by renal involvement, while H&N GPA is thought to be less severe with much lower risk of kidney failure (14). However, recent studies have emphasized that H&N GPA can lead to aggressive, tissue-destructive complications such as saddle-nose, septal perforation, orbital tumor-like mass formation, ear bony wall destruction, and epiglottitis (4–9). H&N GPA is often a chronic-relapsing disorder which in later stages can show higher Vasculitis Damage Indices than sGPA (15, 16). Also, tissue-destructive manifestations encountered in H&N GPA patients are frequently refractory to immunomodulatory therapy, even aggressive immunomodulation with cyclophosphamide and high-dose glucocorticoids (1, 17). Thus, while being localized and identified as “limited” in extent, H&N GPA is an aggressive and tissue-destructive disease subset. Specific cellular mechanisms underlying tissue destruction pathways in patients with H&N GPA have not been described.

Anti-neutrophil cytoplasmic antibodies are a hallmark of GPA and patients with disease dominantly in the head and neck territory mostly produce the anti-PR3 variant (2). In 2009, Kessenbrock et al. reported that neutrophil netting resulting in NET formation, now understood to be a specific type of programed cell death, occurred in patients with GPA (18). NETs are actively released chromatin fibers containing DNA, histones, and neutrophil granule proteins, and are considered a critical component in the pathogenesis of ANCA-associated vasculitis (19, 20). Disordered regulation of NETosis, such as enhanced NET generation as well as reduced NET degradation, and the presentation of NET antigens by specific HLA haplotypes is postulated to play a role in the production of ANCAs (20–22). The interaction of the cell-surface semaphorin 4D, expressed on neutrophils, with plexin B2, expressed on vascular endothelial cells, inhibits NETs generation. This mechanism appears to be impaired in neutrophils of ANCA-associated vasculitis patients, leading to excess NET formation (23). Also, NETs from ANCA-stimulated neutrophils have been reported to induce endothelial cell damage via alternative complement pathway activation with elevated C5a generation (24). Despite the increasing recognition of NETosis in the pathogenesis of ANCA-associated vasculitis, most of the studies are derived from MPO-ANCA associated vasculitis and only few studies of NETosis are available in patients with PR3-ANCA-related GPA (22, 25). Furthermore, although the DNA contained in NETs can activate innate immune cells such as monocytes and dendritic cells via Toll-like receptor (TLR) 9 (26), it remains unknown whether the pathogenic role of NETs in GPA is mechanistically linked to NET-dependent activation of inflammatory cells.

Recent studies have implicated monocytes producing the tissue-destructive matrix metalloprotease (MMP)-9 in breaking the vascular immunoprivilege and enabling vasculitogenic T cells to invade the vessel wall tissue niche (27). In addition to neutrophils, the vascular and extravascular lesions of GPA contain considerable amounts of monocytes (19). Disease activity and PR3-ANCA titers have been correlated with the extent of monocyte activation (28, 29). Serum MMP-9 levels are elevated in GPA patients and MMP-9-producing macrophages are present in GPA tissue lesions (30–32). Taken together, MMPs are suspected to participate in the tissue destruction typical for GPA.

Microbial infection as a key trigger inducing the pathogenomic granulomatous tissue lesions of GPA remains the subject of long-standing debate (33, 34). Increasing evidence suggests a crucial role for the pro-inflammatory anti-microbial S100 family of proteins in driving inflammation in cancers and in rheumatic diseases (35). S100 proteins act as endogenous danger-associated molecular pattern (DAMP) molecules, so-called alarmins (35). By binding to TLR4 or RAGE, S100 proteins are effective amplifiers of inflammatory responses (35). The family members S100A8 and S100A9 originate predominantly from neutrophils and monocytes and are therefore called myeloid-related protein 8 (MRP8) and myeloid-related protein 14 (MRP14). Serum S100A8/A9 concentrations are elevated and associated with disease relapse in PR3–ANCA associated vasculitis (36). Interestingly, S100A9, but not the S100A8/A9 heterodimer, induces pro-inflammatory cytokine production in human mononuclear cells via TLR4 binding and is considered a novel therapeutic target in autoimmune disease (37, 38). S100A9 knockout mice have reduced disease activity in several murine models of inflammatory disease (39–41).

In this study, we have delineated a molecular mechanism for the tissue destructive lesions in patients with H&N GPA. When compared to patients with pulmonary and renal GPA, neutrophils from H&N GPA patients have a much higher propensity to undergo NETosis. These NETs have biological activity as monocytic stimulators and are highly effective in inducing a “tissue-invasive” monocyte phenotype. NETs derived from H&N GPA patients prime monocytes to differentiate into matrix-digestive effector cells by inducing the alarmin S100A9. In a feed-forward loop, S100A9 functions as an inducer of MMP-9, endowing monocytes with proteolytic capabilities and tissue lesions from H&N GPA patients are densely infiltrated by MMP-9-producing macrophages. These data identify the NET-S100A9-MMP-9 axis as a critical component in GPA-related bone, cartilage and connective tissue destruction.

## Methods

### Patients and Controls

The study population included ANCA-positive GPA patients and only patients with active disease were enrolled. Destructive H&N lesions were defined as follows: invasive sinusitis, bone destruction, orbital/skull base involvement, destructive midline disease, severe saddle nose, severe epistaxis, orbital pseudotumor, subglottic stenosis, and persistent otitis media with bone invasion. Patients who had destructive lesions in the ear, eye, nose, or throat were classified as the H&N GPA group. Patients with infectious etiologies, naso-sinal NK/T cell lymphoma and other malignancies were excluded. Patients in the sGPA group all had renal involvement. Demographics of patients are shown in Table 1. Age-gender matched healthy blood donors were obtained from the Stanford Blood Center. They had no history of autoimmune disease, cancer, chronic viral infection, or any other inflammatory syndrome. The study was approved by the Institutional Review Board and written informed consent was obtained as appropriate.

**Table 1:** Patient demographics.

	**H&N GPA (*n* = 22)**	**Systemic GPA (*n* = 24)**	***P*-value**
Age (years, mean ± SEM)	43.1 ± 3.9	47.3 ± 3.4	0.54
Female (*n*, %)	12 (55)	17 (71)	0.36
Disease duration (years, mean ± SEM)	6.0 ± 0.8	7.7 ± 1.1	0.36
ANCA positivity			0.61
PR-3-ANCA (*n*, %)	21 (95)	21 (88)	
MPO-ANCA (*n*, %)	1 (5)	3 (13)	
Treatment at blood sampling			
Prednisolone (*n*, %)	16 (73)	15 (63)	0.75
Cytoxan (*n*, %)	0 (0)	0 (0)	>0.99
Mycophenolate mofetil (*n*, %)	5 (23)	7 (29)	0.74
Azathioprine (*n*, %)	6 (27)	6 (25)	>0.99
Rituximab (*n*, %)	1 (5)	1 (4)	>0.99

### Cells and Culture

PBMCs were isolated from whole blood by Ficoll (STEMCELL Technologies) density centrifugation. Neutrophils were separated from the remaining erythrocyte-rich pellet by dextran sedimentation. Residual erythrocytes were eliminated using red blood cell lysis buffer (BioLegend). CD14^+^ monocytes were cultured in RPMI 1640 medium (Life Technologies) supplemented with 20 ng/mL M-CSF (eBioscience) and 10% FBS (Lonza) for 24 hrs. Monocytes were detached for flow cytometry using StemPro Accutase (Life Technologies).

### Quantitative RT-PCR

Total RNA was extracted with Direct-zol RNA MiniPrep (Zymo Research), and cDNA was reverse transcribed using the High-Capacity cDNA Reverse Transcription Kit (Thermo Fisher Scientific). Gene expression was determined using 2 × SYBR Green qPCR Master Mix (Biotool.com) and a RealPlex2 Mastercycler (Eppendorf). Gene transcript numbers were adjusted relative to β-actin transcripts. Primers used in this study are listed in Table 2.

**Table 2:** List of primers.

**Genes**	**Forward primers**	**Reverse primers**
β-actin	GATCATTGCTCCTCCTGAGC	CGTCATACTCCTGCTTGCTG
S100A8	ATGCCGTCTACAGGGATGAC	ACTGAGGACACTCGGTCTCTA
S100A9	GGTCATAGAACACATCATGGAGG	GGCCTGGCTTATGGTGGTG
MMP-2	AGAAGGATGGCAAGTACGGCTTCT	AGTGGTGCAGCTGTCATAGGATGT
MMP-3	TCCCTCAGGAAGCTTGAACCTGAA	AAACCTAGGGTGTGGATGCCTCTT
MMP-9	TACCACCTCGAACTTTGACAGCGA	GCCATTCACGTCGTCCTTATGCAA
IFNα	ATGCGGACTCCATCTTG	CGTGACCTGGTGTATGAG
IFNβ	GCACTGGCTGGAATGAGACT	TGCTCATGAGTTTTCCCCTGG
IFNγ	ACTAGGCAGCCAACCTAAGCAAGA	CATCAGGGTCACCTGACACATTCA
IL-1β	ATCCAGCTACGAATCTCCGA	CCACTTGTTGCTCCATATCC

### Protein Quantification

Protein quantification of MMP-9 (Abcam, Ab137867) and S100A9 (BD, 565793) by flow cytometry was done using a BD LSRFortessa. Data were analyzed with FlowJo software (Tree Star). For intracellular staining, monocytes were permeabilized using the Cytofix/Cytoperm Kit (BD).

### NETosis Assay and NET Isolation

Isolated neutrophils were cultured for 1.5 h to quantify spontaneous NETosis. NETosis was induced in isolated neutrophils with PMA (20 ng/ml) in RPMI 1640. To detect NETosis, cells were stained with SYTOX green (Thermo Fisher) and analyzed by flow cytometry. Alternatively, neutrophils were seeded on a PLL-coated 96-well glass-bottom plate, treated with PMA and SYTOX green and staining was analyzed by confocal microscopy (LSM 710, Carl Zeiss). To analyze DNA-NET structures, neutrophils in PLL-coated 96-well glass-bottom plates were stained with propidium iodide (PI) and imaged by confocal microscopy. Image analysis and software-based automated quantification of the DNA area surrounding netting neutrophils was executed by “the DNA Area and NETosis Analysis” (DANA) software tool plug-in for Fiji/ImageJ (42). NETs were isolated based on published protocols (43). Briefly, neutrophils were cultured on a 6-well plate and stimulated with PMA. In a multi-step centrifugation process, we first gained a cell-free and NET-rich solution, from which we then pelleted DNA with a microcentrifuge. DNA concentrations were measured using a spectrophotometer (NanoDrop Technologies) and normalized to cell numbers as indicated.

### NETs and Monocyte Coculture

CD14^+^ monocytes were isolated from healthy individuals and were treated with isolated NETs (5 μg/ml) derived from patients with H&N GPA, patients with sGPA or age-matched healthy controls. In some experiments, isolated NETs from patients with H&N GPA were treated with DNase (Zymo Research) to degrade DNA for 15 min before being added to monocytes. Recombinant S100A9 (100 ng/ml) (R&D Systems, 9254-S9-050) or paquinimod (50 or 100 μM) (MedKoo, 319595) were used to treat monocytes for 24 h.

### “Invasion” Assay

8.0 μm Millicell culture plate inserts (PI8P01250, Millipore Sigma) were fitted into 12-well plates. A collagen gel was placed into the insert, which was composed of 1 mg/ml of collagen type IV (CC076, Sigma-Aldrich), and 2.1 mg/ml collagen type I (5005-100ML, AdvancedBioMatrix) as previously described (27). 100,000 monocytes pre-treated with isolated NETs (5 μg/ml) were layered on top of the collagen. The matrix-invasive capacity of monocytes was quantified by enumerating the cells in the lower chamber after 24 h. For MMP-blocking experiments, monocytes were placed on top of the collagen gel and anti-MMP-9 antibody (10 μg/mL) (GS-5745, Gilead Sciences) or isotype control IgG was added. Cells that had passed through the gel were counted after 24 h.

### Immunohistochemistry

Dual-color tissue staining was performed to detect the macrophage marker PU.1 and MMP-9 as previously described (27). PU.1 (BD Pharmingen) was used at a 1:100 dilution for 60 min, followed by substrate incubation with Vector substrate (SK-4705) for 15 min. Next, tissue sections were incubated with anti-pro-MMP-9 (Abcam) at a 1:300 dilution for 50 min, followed by Vector substrate (SK-4200) for 15 min.

### Statistical Analysis

Data were analyzed using GraphPad Prism software (Version7; GraphPad Software, La Jolla, CA). Statistical significance was assessed by paired *t-*test or one-way analysis of variance (ANOVA) with *post-hoc* Tukey's multiple comparisons test (3 or more groups). A two-sided *P*-value of < 0.05 was considered significant.

## Results

### Neutrophils From Patients With Head and Neck GPA Are Prone to NETosis

**Figure 1:**
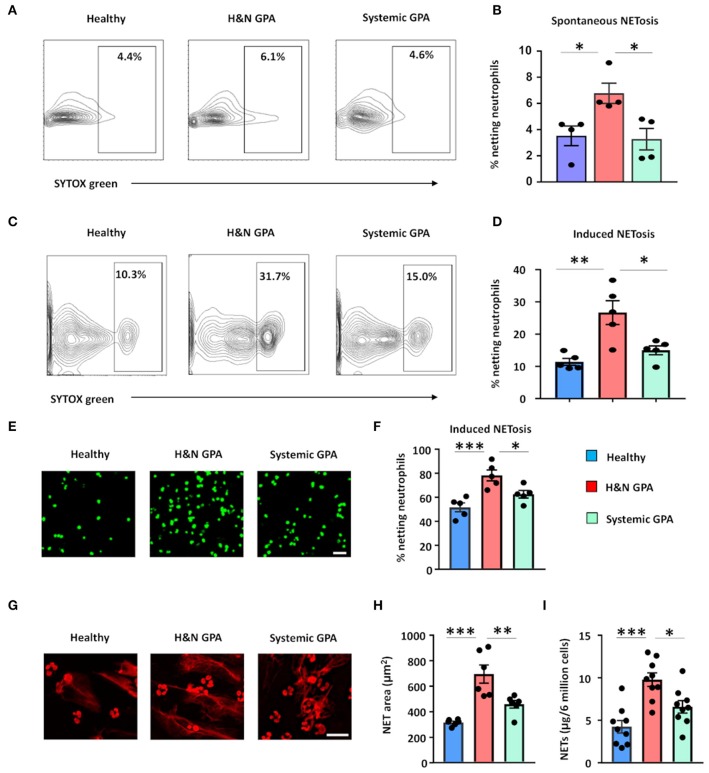
Neutrophil NET formation in patients with GPA correlates with clinical disease pattern. Blood was collected from patients with H&N GPA, systemic GPA, and age-matched healthy individuals. NET formation in isolated neutrophils was assessed by three methods. **(A,B)** Neutrophils were cultured for 1.5 h and spontaneous netting neutrophils were assessed as SYTOX® Green-positive cells by flow cytometry. Representative results **(A)** and data from four experiments **(B)**. **(C,D)** Neutrophils were treated with phorbol myristate acetate (PMA; 20 ng/ml) for 1.5 h. Netting neutrophils were detected as SYTOX® Green-positive cells by flow cytometry. Representative results **(C)** and data from five experiments **(D)**. **(E,F)** Neutrophils were activated with PMA (20 ng/ml) for 4 h and stained with SYTOX® Green. Netting neutrophils were identified by confocal microscopy. Representative images (scale bar = 100 μm) **(E)** and results from five experiments **(F)**. **(G,H)** Netting was induced with PMA treatment (20 ng/ml; 4 h) and extracellular DNA fibers were stained by propidium iodide (PI). Representative images (scale bar = 50 μm) **(G)** and results from six experiments **(H)**. **(I)** Measurement of DNA content in isolated NETs derived from 6 million PMA-treated neutrophils (20 ng/ml; 4 h), quantified spectrophotometrically from nine patients with H&N GPA, nine patients with systemic GPA, and nine healthy individuals. All data are mean ± SEM. **(B,D,F,H,I)** One-way ANOVA with *post-hoc* Tukey's multiple comparisons test. *P < 0.05; **P < 0.01; ***P < 0.001.

Patients with GPA have been reported to generate NETs more efficiently than healthy individuals (22, 25). To examine whether differences in clinical patterning are correlated with the ability to release extracellular DNA, we tested the capacity of neutrophils to undergo NETosis in two patient cohorts: (1) patients with sGPA and (2) patients with H&N GPA. We quantified neutrophil extracellular DNA release by several different methods: detection of SYTOX® Green-positive netting neutrophils using flow cytometry and fluorescence microscopy, quantification of extracellular DNA fibers by propidium iodide staining, and quantification of extracellular DNA measuring SYTOX® Green-positive cell-appendant DNA (44). First, we assessed spontaneous NETosis in unstimulated neutrophils by staining with SYTOX® Green. Neutrophils isolated from H&N GPA patients underwent NETosis at a higher rate than neutrophils from sGPA patients and healthy individuals (Figures 1A,B). Next, freshly isolated neutrophils from healthy control subjects and H&N and sGPA patients were stimulated by phorbol myristate acetate and stained by SYTOX® Green (Figures 1C,D). Flow cytometric analysis showed that neutrophils from both H&N GPA and sGPA patients underwent NETosis with higher frequency than those from healthy individuals. However, neutrophils from H&N GPA were particularly efficient in extracellular DNA release (Figures 1C,D). Confocal microscopy confirmed higher rates of NETosis in H&N GPA compared to sGPA and healthy controls (Figures 1E,F).

Next, we visualized extracellular chromatin fibers formed by netting neutrophils characterized as extracellular DNA networks around GPA neutrophils (Figures 1G,H). To quantify NET-structures, we used a software-based tool that allows automated quantification of NETosis-derived DNA structures (DNA Area and NETosis Analysis; DANA) (42). DANA software calculated the area of DNA-NET-structures to be greater around neutrophils from patients with H&N GPA than around neutrophils from sGPA patients and control neutrophils (Figures 1G,H).

Finally, we isolated NETs (43) and quantified the amount of DNA contained within these structures. Isolated NETs from H&N GPA patients had a higher DNA content compared to control neutrophils and neutrophils from sGPA patients (Figure 1I).

These results indicate that NET formation differs in clinical subsets of GPA patients and that neutrophils from patients with H&N GPA are particularly efficient in extracellular DNA release.

### NETs Are Recognized by Monocytes and Induce Expression of the Alarmin S100A9

**Figure 2:**
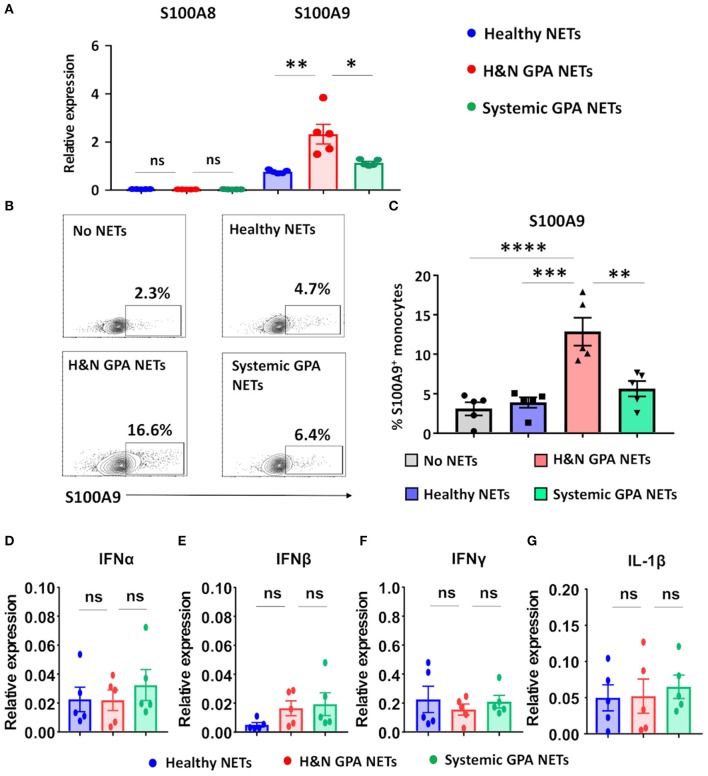
Neutrophilic NETs induce the alarmin S100A9. Neutrophilic NETs were generated from H&N GPA, systemic GPA, and age-matched healthy individuals and added to CD14^+^ monocytes (5,000 ng/ml) for 24 h. Transcript expression was quantified by RT-PCR. **(A)** Transcripts for the alarmins S100A8 and S100A9 (*n* = 5, each group). **(B,C)** Intracellular expression of S100A9 protein measured by flow cytometry. Representative contour plots **(B)** and summary results from five experiments **(C)**. **(D–G)** Transcript expression for IFNα, IFNβ, IFNγ, and IL-1β (*n* = 5, each group). All data are mean ± SEM. **(A,C)** One-way ANOVA with *post-hoc* Tukey's multiple comparisons test. *P < 0.05; **P < 0.01; ***P < 0.001; ****P < 0.0001; ns *P* > 0.05.

In the tissue lesions of GPA, the initial neutrophil-rich infiltrates are gradually replaced by monocytes, creating a microenvironment in which monocytes encounter dying neutrophils (19). We tested the hypothesis that nucleic acids released into the extracellular space represent danger-associated patterns and function as pro-inflammatory stimuli by triggering the production of alarmins (26, 35). Freshly isolated monocytes were treated with isolated NETs from three sources: patients with H&N GPA, patients with sGPA, and age/gender matched healthy controls. NET-treated monocytes promptly upregulated transcription of the alarmin S100A9. H&N GPA-derived NETS were the strongest inducer of S100A9, inducing transcript levels more than two-fold higher than sGPA and control-derived NETs (Figure 2A). In contrast to S100A9, the S100 family member S100A8 was unaffected by the NETs. Transcriptome analysis was confirmed by assessment of S100A9-producing monocytes by flow cytometry (Figures 2B,C). S100A9-staining monocytes were infrequent, but following stimulation with H&N GPA NETs, 10–15% of monocytes became S100A9 producers. NETs obtained from healthy control neutrophils or from sGPA neutrophils both lacked the ability to trigger S100A9 production in monocytes (Figures 2B,C). We tested whether NETs from the different study cohorts were able to induce interferons (IFN) and whether interleukin (IL)-1β was part of the monocytic response to NET recognition (Figures 2D–G). We found that transcripts for IFNα, IFNβ, IFNγ, and IL-1β were unaffected by NETs.

These data identify NETs as effective monocyte stimulators, providing signals that upregulate endogenous DAMPs, such as S100A9, thus promoting innate immune responses.

### Induction of S100A9 Requires Recognition of DNA

**Figure 3:**
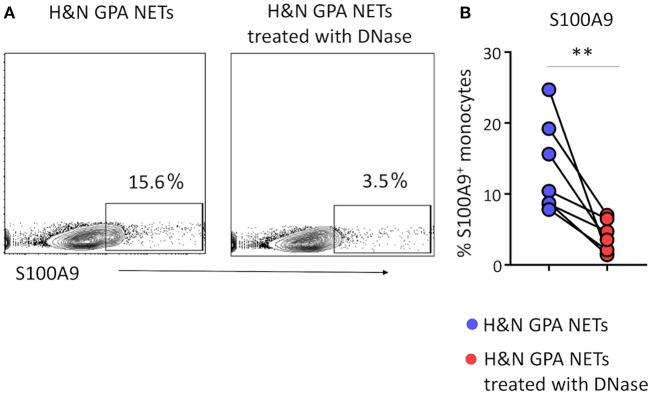
Induction of S100A9 requires recognition of NET DNA. Neutrophilic NETs isolated from patients with H&N GPA were treated with DNase for 15 min. Monocytes were stimulated with treated or untreated NETs for 24 h. S100A9 protein expression in monocytes was analyzed by flow cytometry. Representative contour plots **(A)** and data from seven experiments **(B)**. Paired *t*-test. **P < 0.01.

Extracellular DNA has been reported to interact with pattern recognition receptors, such as Toll-like receptors (TLRs) to participate in intercellular communication (24). To test whether the nucleic acids contained in NETs provide monocyte-activating signals, we treated purified NETs with DNase prior to coculture with monocytes and monitored S100A9 expression as the outcome parameter. Digestion of the DNA in NETs minimized the frequencies of S100A9-producing monocytes (Figures 3A,B). Thus, DNA expulsed from neutrophils actively participates in directing monocyte effector functions.

### S100A9 Regulates MMP-9 Production in Monocytes

**Figure 4:**
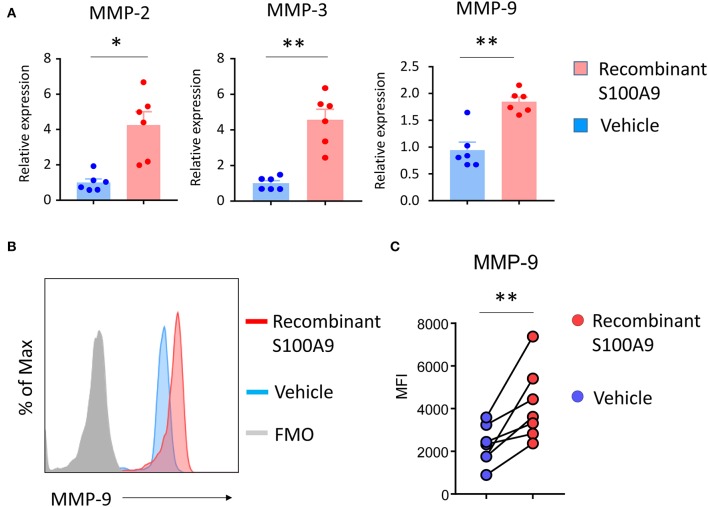
S100A9 upregulates monocyte MMP production. Purified monocytes were treated with recombinant S100A9 (100 ng/ml) or vehicle for 24 h. Transcripts for MMP-2, MMP-3, and MMP-9 were quantified by RT-PCR (*n* = 6) **(A)**. Intracellular MMP-9 protein expression was analyzed by flow cytometry **(B,C)**. Representative histograms **(B)** and data from seven experiments **(C)**. Paired *t*-test. *P < 0.05, **P < 0.01.

S100A9 has been reported to act as a cell-to-cell mediator, being secreted from monocytic cells and regulating the production of pro-inflammatory effector molecules and chemokines in neighboring cells. TLR4 and RAGE have been identified as S100A9 receptors (35). A hallmark of the monocyte-occupied GPA tissue lesions are metalloproteinase-producing effector cells and MMP-2, -3, and -9 are known to be elevated in the serum of GPA patients (30–32). We hypothesized that the excessive S100A9 production triggered by DNA-containing NETs may amplify tissue inflammation by interfering with the regulation of MMP production. To test this hypothesis, we treated healthy monocytes with recombinant S100A9. Transcript analysis for MMPs gene (MMP-2, MMP-3, and MMP-9) showed that gene expression of MMP-2, -3, and -9 were strongly enhanced by recombinant S100A9 (Figure 4A). Flow cytometric analysis confirmed that recombinant S100A9 was sufficient to double the MMP-9 load of monocytes (Figures 4B,C).

This data placed S100A9 upstream of metalloproteinase production, a major effector function of tissue-residing monocytes and macrophages.

### NET-Induced S100A9 Functions as a MMP-9 Inducer

**Figure 5:**
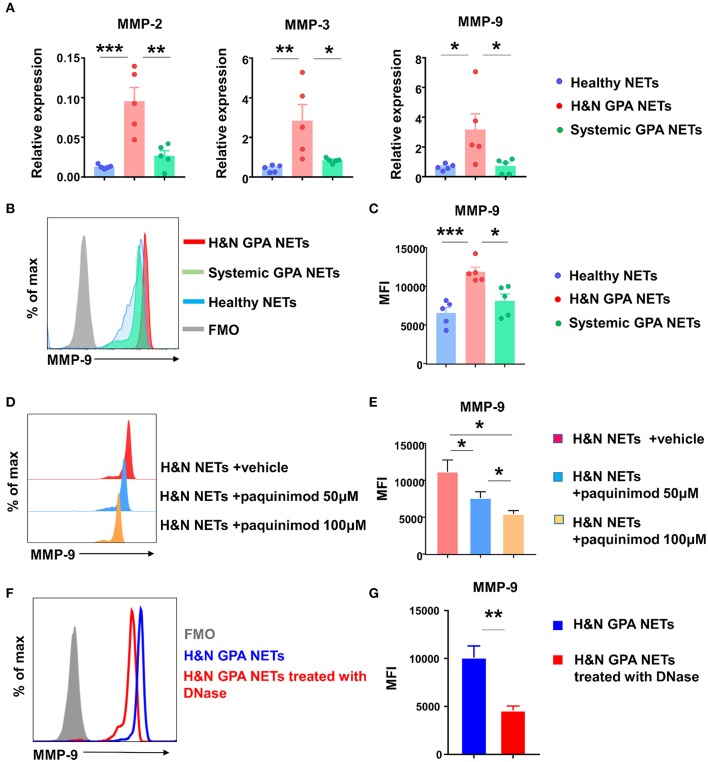
NETs-induced S100A9 promotes MMP-9 production. NETs were induced from neutrophils of GPA patients and matched controls. Monocytes were treated with isolated NETs for 24 h. **(A)** Transcripts for MMPs were assessed by RT-PCR. **(B,C)** Flow cytometrical quantification of intracellular MMP-9 protein. Representative histograms **(B)** and MFIs from five experiments **(C)**. **(D,E)** S100A9 receptor binding was blocked with paquinimod in NET-monocyte cocultures. MMP-9 protein was quantified by flow cytometry as in **(B)**. Representative histograms **(D)** and results from six experiments **(E)**. NETs from H&N GPA patients were treated with DNase for 15 min and added to monocytes for 24 h. Intracellular MMP-9 expression was examined using flow cytometry. Representative histogram **(F)** and MFIs from six experiments **(G)**. All data are mean ± SEM. **(A,C,E)** One-way ANOVA with *post-hoc* Tukey's multiple comparisons test. **(G)** Paired t test. *P < 0.05; **P < 0.01; ***P < 0.001.

Given that recombinant S100A9 is a potent regulator of monocytic MMP-9, we asked the question whether recognition of NETs culminated in the induction of MMP-9, thus directly affecting monocyte/macrophage effector functions. First, we isolated NETs from neutrophils purified from patients with H&N GPA, patients with sGPA, and healthy controls, placed them on monocytes and quantified transcripts for the three major MMPs, MMP-2, MMP-3, and MMP-9 (Figure 5A). MMP transcripts from cultures with healthy NETs and sGPA NETs were indistinguishable. In contrast, interaction with NETs harvested from H&N GPA resulted in strong upregulation of MMP-2, MMP-3, and MMP-9. Flow cytometry confirmed that monocytes pre-treated with H&N GPA NETs contained higher amounts of MMP-9 protein (Figures 5B,C).

Alarmins mediate intercellular communication by binding to TLRs. We explored whether NET-induced S100A9 regulates MMP-9 production through TLR4 recognition. The quinolone-3 carboxamide derivative paquinimod (ABR-215757), binds to S100A9, but not to the S100A8/9 complex or to S100A8, and inhibits S100A9-binding to TLR4 and RAGE (38, 45, 46). We included paquinimod in the NET-monocyte cocultures and measured the effect on MMP-9 induction. Paquinimod effectively prevented MMP-9 induction in a dose-dependent manner (Figures 5D,E).

To address the possibility that MMP-9 induction was facilitated by S100A9 contained within the neutrophilic NETs and did not require NET-dependent monocyte activation, we pretreated the NETs with DNase and assessed the dependence of MMP-9 induction on intact NET DNA (Figures 5F,G). MMP-9 production was minimal after DNase pretreatment, confirming that NET proteins were insufficient to activate protease-producing macrophages and that the pathogenic cascade required DNA recognition.

These data established a NET-S100A9-TLR4-MMP-9 signaling cascade and directly implicated extracellular DNA in the regulation of monocyte effector functions.

### NET-Activated, MMP-9-Producing Monocytes Are Tissue Invasive

**Figure 6:**
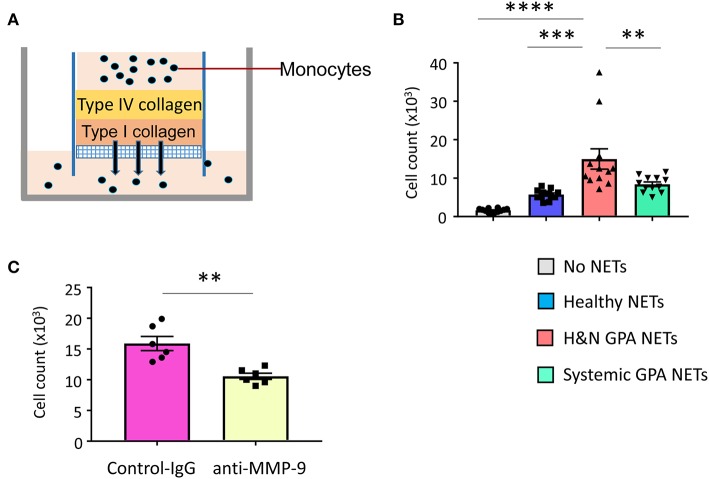
NET-stimulated monocytes are tissue invasive. Basement membrane matrix was built by coating collagen IV and collagen I layers on top of porous membranes. 100,000 monocytes which were untreated or pre-treated with purified NETs were layered on top of the collagen matrix **(A)**. Invasive capacity of monocytes was quantified by enumerating the cells in the lower chamber after 24 h. Mean ± SEM from 12 experiments **(B)**. NET-stimulated monocytes were incubated with anti–MMP-9 antibody (10 μg/mL) or isotype control IgG. Cells that had penetrated through the matrix layers were counted after 24 h. Mean ± SEM from six experiments **(C)**. One-way ANOVA with *post-hoc* Tukey's multiple comparisons test **(B)**. Paired *t*-test **(C)**. **P < 0.01; ***P < 0.001; ****P < 0.0001.

MMP-9, a type IV collagenase and gelatinase, is critically involved in extracellular matrix remodeling and is required for the migration of neutrophils and monocytes across the basement membrane (27, 47). Since H&N manifestations of GPA are characterized by tissue destruction, including erosion of bone and cartilage, we explored whether NET-activated monocytes have tissue-invasive features. We assembled an artificial collagen-gel to resemble tissue matrix and tested whether monocytes could digest and penetrate through the gel (Figure 6A). Measurements of matrix-invasive capacity of monocytes exposed to control and patient-derived NETs revealed that H&N GPA-derived NETs were able to modulate monocyte function, rendering them tissue invasive (Figure 6B). To examine whether the matrix-digestive phenotype was dependent on MMP-9, we applied a specific MMP-9-inhibitor (GS-5745). Treatment with the MMP-9-inhibitor led to a significant reduction in the number of monocytes treated with H&N GPA-derived NETs that penetrated through the type IV/type I collagen matrix (Figure 6C).

**Figure 7:**
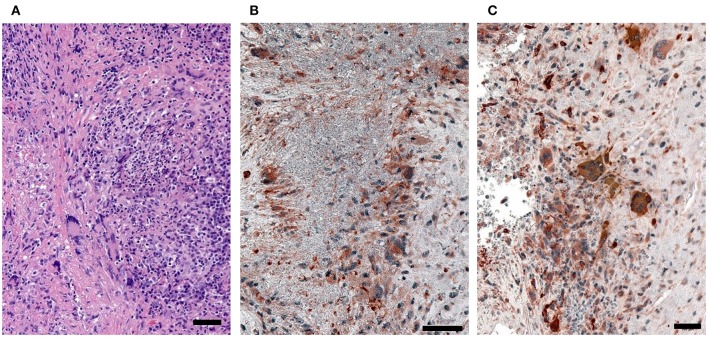
MMP-9-producing macrophages in GPA tissue lesions. Tissue biopsies were collected from sinal and nasal lesions of patients with H&N GPA. **(A)** H&E (hematoxylin and eosin)-stained sections. **(B,C)** Dual-color immunostaining for the macrophage marker PU.1 (nuclei dark gray) and pro-MMP-9 (red). (scale bar = 100 μm).

To assess the *in vivo* relevance of tissue-invasive MMP-9-producing macrophages, we performed immunohistochemical staining in tissue biopsies from patients with H&N GPA (Figure 7). In tissue sections collected from naso-sinal lesions, cells staining double-positive for the macrophage marker PU.1 and pro-MMP-9 densely populated the tissue infiltrates (Figures 7B,C). Essentially, every PU.1-positive macrophage produced pro-MMP-9. Notably, multinucleated giant cells stained strongly positive for the metalloproteinase (Figure 7C). The majority of neutrophils were found within the necrotizing granulomatous lesions and were placed in close proximity to pro-MMP-9^+^ macrophages and multinucleated giant cells (Figure 7); co-localizing the NET producers and signal-receiving macrophages. Pro-MMP-9 staining was rarely encountered within neutrophils, identifying macrophages and multinucleated giant cells as the major cellular source of tissue-injurious MMP-9.

Together, these data provided a mechanistic link between NET recognition, MMP-9 induction and tissue-destructive capabilities.

## Discussion

**Figure 8:**
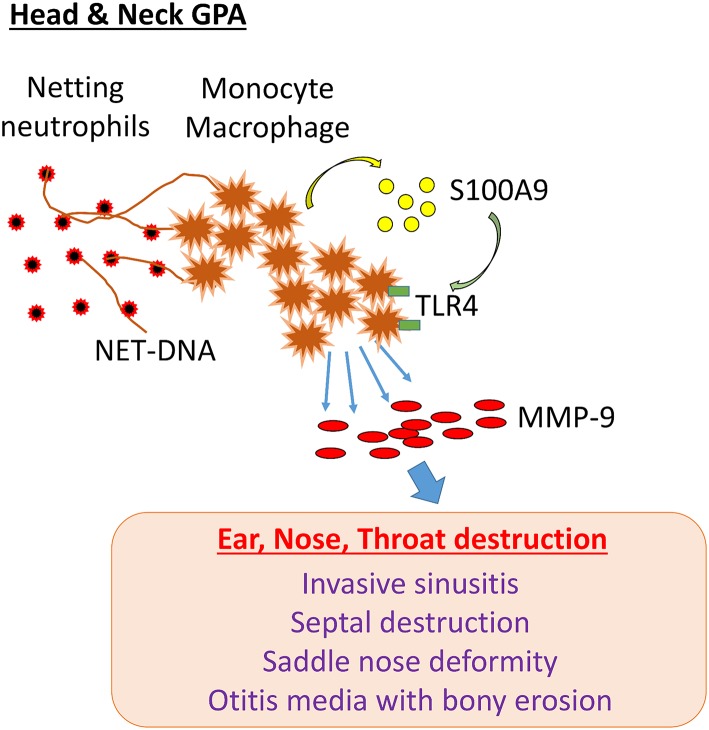
NET-induced pathology in H&N GPA. In patients with GPA dominated by head and neck manifestations (H&N GPA), neutrophils are highly susceptible to NETosis. NET-DNA is recognized by monocytes and macrophages and induces the alarmin S100A9. In a feed-forward loop, S100A9 binds to TLR4 and triggers the induction of the protease MMP-9. MMP-9-producing monocytes/macrophages have tissue-invasive and destructive capabilities. The pathway correlates with distinct clinical manifestations: invasive sinusitis, bony erosions of the sinal walls, septal perforation, saddle nose deformity, otitis media with bony invasion, invasive growth of orbital pseudotumor.

GPA patients with disease dominated by head and neck manifestations are often classified as having “limited GPA,” but their clinical presentation is characterized by irreversible damage of bone, cartilage, and other tissue compartments. GPA lesions lead to saddle nose deformity, invasive sinusitis with destruction of the sinus walls, septal perforation, orbital tumor-like mass formation, bony erosion in the middle ear and mastoid, trachea- and bronchomalacia due to cartilage resorption and epiglottis. GPA-related tissue damage in the head and neck can cause permanent disability due to the loss of vital functions. Here, we have identified a cascade of pathogenic events that begins with the release of neutrophil DNA and ends with the formation of tissue-digestive monocytes/macrophages. The molecular elements in this pathogenic cascade involve the recognition of extracellular DNA by monocytes, induction and secretion of the alarmin S100A9, autocrine triggering of TLR4 and upregulation of MMP-9 (Figure 8). Essentially, NETs drive an S100A9-dependent feed-forward mechanism that generates matrix destructive monocytes. By linking into endogenous danger signaling, NETs exhibit strong immunomodulatory abilities, turning protective immunity into tissue-damaging immunity. The NET-S100A9-TLR4-MMP-9 axis provides new opportunities for immunosuppressive strategies in GPA (Figure 8).

Neutrophils utilize the release of DNA into the extracellular space to trap and kill invading pathogens. In the setting of GPA, NETs are considered to be a source of self-molecules that potentially promote autoimmunity as dendritic cells are able to take up NET-components, present autoantigens and thus promote autoantibody production. The present study implicates NETs into immunomodulatory functions beyond autoantibody production by defining a role of NETs in regulating innate immunity. Specifically, we delineated a pathway through which neutrophils can direct the activity and the effector differentiation of monocytes. We identified a striking upregulation of MMP-9 in monocytes which recognized NETs derived from H&N GPA patients. Mechanistically, tissue-destructive monocytes were induced through endogenous S100A9 production, elicited by the interaction of extracellular DNA with monocytes. Blocking experiments with the S100A9 inhibitor paquinimod drew attention to S100A9 binding to TLR4. TLR4-dependent activation of NF-κb has previously been reported to drive MMP-9 production (48). Paquinimod also inhibits the interaction of S100A9 with RAGE. Further studies are needed to define the details of S100A9-dependent MMP-9 induction, as every step in the cascade is a potential therapeutic target to prevent the emergence of tissue-destructive monocytes and macrophages in GPA tissue lesions.

In the present study, S100A9 but not S100A8 was sufficient to induce tissue-invasive MMP-9-producing monocytes. S100A8 and S100A9 are thought to be less stable as homodimers than heterodimers. However, recent studies have clarified that heterodimer formation is not an absolute requirement. S100A9, particularly when generated under inflammatory conditions, was functional as a protease-resistant stable homodimer (37, 38, 49). S100A9^−/−^ mice have been reported to develop reduced tissue inflammation (39–41) and S100A9-blocking antibody ameliorates collagen-induced arthritis (50). Paquinimod binds S100A9 but not S100A8 or the S100A8/A9 heterodimer, blocking the interaction with both TLR4 and RAGE (38). Here, paquinimod was sufficient to block NET-induced MMP-9 production in monocytes, suggesting that S100A9 alone is sufficient as an inflammatory amplifier in GPA.

MMP-9 is thought to demolish not only matrix and basement membrane but also bone and cartilage (51–54). Recent proteomic studies have identified MMP-9 as a disease activity marker in ANCA-associated vasculitis, but not in patients with rheumatoid arthritis or systemic erythematosus (55). Also, MMP-9-producing macrophages are part of the tissue infiltrate in GPA nasopharyngeal mucosa lesions (30–32). Furthermore, MMP-9 expression is elevated and closely correlated with the recurrence and severity of chronic sinusitis and nasal polyps, particularly in naso-sinal bone lesions (56–58), in line with the preponderance of NET-induced MMP-9 in H&N GPA patients. Future prospective studies examining serum MMP-9 levels as a promising disease biomarker for chronic-relapsing H&N GPA will be of great clinical interest. NETs have been implicated in driving interferon responses (59) and the current study adds additional pathways to the pathogenic effects of neutrophil-derived DNA. Our data provide strong support for the hypothesis that there are not only quantitative differences but also qualitative differences in the neutrophilic NETs produced by patients with distinct clinical manifestations.

Recognizing the NET-S100A9-MMP-9 axis as a disease pathway in GPA provides opportunities for novel therapeutic strategies. Blocking S100A9 using paquinimod or anti-MMP-9 antibodies has been reported to modify the disease course in both murine models and in clinical trials. This is of particular importance for patients with H&N GPA, who despite the limited territory of disease involvement, suffer from disabling complications and often are refractory to conventional immunosuppressive interventions.

## Data Availability Statement

The data which support the findings of the study are available from the corresponding author upon reasonable request.

## Ethics Statement

The studies involving human participants were reviewed and approved by Stanford University Institutional Review Board. The patients/participants provided their written informed consent to participate in this study.

## Author Contributions

CW, MA, MZ, and JG designed the study and analyzed the data. MA and MZ performed the experiments. MA, MZ, NI, SO, and PH analyzed clinical data. GB provided expertise in tissue analysis and case identification. CW, MA, GB, and JG wrote the manuscript.

### Conflict of Interest

The authors declare that the research was conducted in the absence of any commercial or financial relationships that could be construed as a potential conflict of interest.
